# A rare case of primary breast angiosarcoma in a male: a case report

**DOI:** 10.1186/s12885-018-4895-3

**Published:** 2018-10-16

**Authors:** Benedito Borges da Silva, Walberto Monteiro Neiva Eulálio Filho, Pedro Vitor Lopes Costa, Rozirene Araújo Silva, Ariton Mendes Conde Junior, Diego Cipriano Chagas, Mariella de Almeida Melo, Fidelis Manes Neto, Cleciton Braga Tavares, Edilson Carvalho de Sousa Júnior, Eid Gonçalves Coelho, Viriato Campelo, Luis Henrique Gebrim, Raimundo Gerônimo da Silva Junior

**Affiliations:** 1 Postgraduate Program of the Northeast Network of Biotechnology (RENORBIO), Federal University of Piauí, Teresina, Brazil; 2 Postgraduate Program in Health Sciences, Federal University of Piaui, Teresina, Brazil; 3 Getúlio Vargas Hospital, Federal University of Piauí, Elias Joao Tajra Avenue, 1260, CEP, Teresina, Piauí 64049-300 Brazil

**Keywords:** Male, Breast, Angiosarcoma

## Abstract

**Background:**

Sarcomas account for less than 1% of primary breast cancers, and breast angiosarcomas are responsible for only 0.05% of all breast malignancies. The male breast has the same potential for malignant transformation as the female breast. However, due to anatomical differences in the breast and the low incidence of angiosarcoma, it is difficult to determine how male breasts can be affected by this type of tumor.

**Case presentation:**

A 36-year-old male patient was admitted to the hospital with a palpable lump in his right breast. Lymphadenopathy was negative. Ultrasonography showed a hypoechoic mass with partially defined contours, measuring 4.0 × 3.0 cm, with muscle infiltration. Histological examination revealed a malignant tumor. Radical mastectomy was then performed with clear surgical margins. The patient began chemotherapy with paclitaxel. Following the second cycle of chemotherapy, he presented with headache and seizures due to a frontal lobe metastasis. Twenty days after the onset of neurological symptoms, the patient died.

**Conclusions:**

Primary angiosarcomas of the male breast are extremely rare. This is the sixth case published in the literature. It is in agreement with other studies in the literature concerning clinical presentation and poor prognosis. Treatment consists in surgical removal of the tumor with clear margins and without axillary lymphadenectomy.

## Background

Soft tissue sarcomas are a heterogenous group of malignant neoplasms derived from mesenchymal cells. Sarcomas make up approximately 1% of malignant tumors in adults [1]. Angiosarcoma is a rare subtype of sarcoma with a poor prognosis that originates from vascular or lymphatic endothelial cells. The annual incidence of angiosarcoma is 2–3 cases per million inhabitants. Around 100 cases are diagnosed in the United States per year, accounting for 1–5% of all soft tissue sarcomas [2].

Nevertheless, breast sarcomas account for less than 1% of primary breast cancers [3]. Breast angiosarcomas, in turn, account for only 0.05% of malignant breast neoplasms. Primary and secondary angiosarcomas have a poor prognosis. Secondary angiosarcomas usually have an important causal link with previous radiation exposure [4]. Due to the low incidence of angiosarcoma, it is difficult to determine how the male breast is affected by this type of tumor. After extensive literature review, only 5 cases of primary angiosarcoma of the male breast were found [5–9], which justified the current case presentation.

## Case presentation

**Fig. 1:**
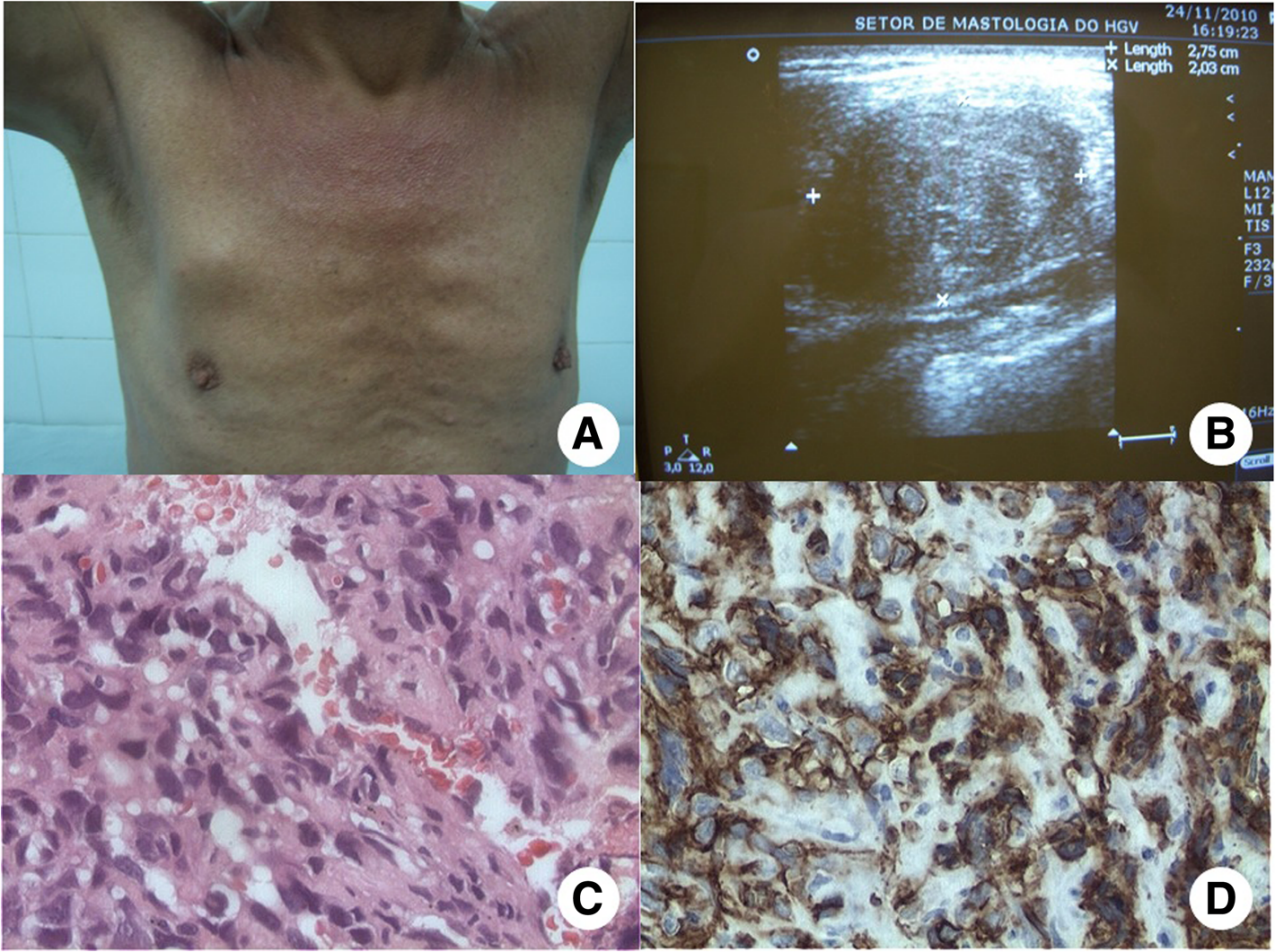
Tumor located at the upper region of the right breast in a male (**a**). Hypoechoic mass with partially defined contours, measuring 4.0 × 3.0 cm at the upper region of the pectoralis major muscle at the 12 o’clock position on ultrasonography (**b**). Poorly differentiated angiosarcoma (Histopathology, HE staining of 400×) (**c)**. Immunohistochemical study showed positivity for CD31 endothelial marker (**d**)

A 36-year-old Brazilian male patient was admitted to the hospital with a palpable lump in his right breast, located at the junction of the upper quadrants of the right breast (Fig. 1a). On physical examination, the lesion appeared firm with irregular margins. Axillary lymphadenopathy was negative and there were no palpable supraclavicular nodes. On breast imaging, ultrasonography showed a hypoechoic mass with partially defined contours measuring 4.0 × 3.0 cm, located at the upper region of the right pectoralis major muscle at the 12 o’clock position with muscle infiltration (Fig. 1b). Histological examination of core biopsy samples revealed a malignant tumor. Preoperative exams, such as X-rays and chest CT scan, abdominal US did not show any signs of disease. Radical mastectomy was then performed, due to pectoralis major muscle infiltration, consisting in removal of the breast along with the major and minor pectoralis muscles. Biopsy of the sentinel lymph node was performed. Gross examination revealed a solid tumor measuring 3.7 × 3.5 cm with a yellowish-tan cut surface and local foci of hemorrhage. Histopathology showed intravascular papillary proliferation of endothelial cells, spindle cell areas and necrosis, atypia and prominent mitotic figures, consistent with the diagnosis of high-grade angiosarcoma with areas of infiltration of the pectoralis major muscle (HE staining, magnification of 400×) (Fig. 1c). Histopathology also demonstrated a surgical specimen with clear margins, absence of angiolymphatic and perineural invasion, in addition to sentinel lymph node free of metastasis. Immunohistochemical study revealed a tumor positive for CD31 marker (Fig. 1d), confirming the vascular nature of the tumor. At the two-week follow-up of the surgical procedure, adequate wound healing was observed, without any evidence of the disease. The patient was transferred to the clinical oncology department, where he presented with severe headache and seizures after the second cycle of adjuvant chemotherapy with paclitaxel. Magnetic resonance imaging of the brain was ordered, revealing a right frontal parasagittal lesion, measuring 1.3 × 1.1 cm with a hemorrhagic component and perilesional edema, suggestive of brain metastasis. The disease progressed rapidly, culminating in the patient’s death at 20 days after the onset of neurological symptoms.

## Discussion

Breast sarcoma is a rare malignant neoplasm with a poor prognosis. Similar to other breast malignancies, the incidence of breast sarcomas in females is much higher than in males. Due to the low incidence of these sarcomas, it is difficult to carry out prospective studies to define therapeutic strategies. As a result, treatment is based on small case series or small retrospective studies. Sarcomas are classified into diverse subtypes. The most common subtypes are angiosarcomas and pleomorphic sarcomas, accounting for approximately 50% of cases [5, 10].

Breast angiosarcomas are extremely rare malignancies that are responsible for only 0.05% of all malignant breast tumors [4]. Knowledge of this type of malignant neoplasm is limited. Major studies are currently based on retrospective cohorts obtained from national databases. Despite difficulty in studying angiosarcoma, patient prognosis is poor, and survival rates are inferior to those of ductal breast carcinomas [11]. Risk factors for the development of secondary angiosarcomas include a well-demonstrated causal relationship with radiotherapy exposure [12]. Furthermore, viral infections have been implicated in tumor pathogenesis, although strong evidence is lacking [13]. It is known that the male breast has the same potential for malignant transformation as the female breast. However, the incidence of breast tumors in males is less than 1% of the total breast tumor cases in females [14]. Primary angiosarcomas of the male breast are extremely rare, with only 5 cases published in the literature [5–9].

Angiosarcomas may be classified as primary or secondary. Primary breast tumor usually manifests itself as a rapidly growing unilateral or bilateral breast lump, commonly occurring in women in their thirties or forties. Secondary breast tumor usually manifests itself as a skin lesion following radiotherapy for conservative breast cancer treatment [9, 15]. The differential diagnosis of primary tumors should include fibrosarcoma, myxoid sarcoma, liposarcomas, phyllodes tumor, histiocytomas, benign hemangiomas and angiomatosis [16]. Initial studies include breast mammography and/or ultrasonography to characterize the lesion. Definitive diagnosis is confirmed by histopathologic analysis after core needle biopsy, however surgical biopsy is acceptable in the absence of core needle biopsy [10].

Angiosarcomas metastasize preferentially by the hematogenous route, and the lung is the most common site of metastasis [17–19], nevertheless some authors have shown an incidence of lymph node metastases up to 6.1% [20]. The median survival rate of patients with primary angiosarcoma of the breast ranges from 1 to 2 years [5]. The presence of distant metastasis is the major contributing factor for poor patient prognosis [11], as occurred in the present case, whose patient died 20 days after experiencing symptoms of brain metastasis.

The mainstay of treatment for angiosarcoma is surgical tumor excision with clear margins. Axillary lymph node resection is not recommended due to the low rate of lymph node metastasis, however due to a possibility of lymph node metastases up to 6.1% [20], sentinel lymph node biopsy was performed in the present case. Surgery may be complemented with adjuvant radiotherapy and chemotherapy for high-grade tumors, although the efficacy of either technique is still not clear [4, 5, 9, 11]. In the present case, radical surgery was performed and adjuvant chemotherapy with paclitaxel was initiated, due to the high histologic grade of the tumor and pectoralis major muscle infiltration, although some authors have shown that the schedule with doxorubicin plus ifosfamide was associated with improved of progression free survival compared to anthracycline [21]. As reported in the current case, primary angiosarcomas of the male are extremely rare and highly aggressive. To the best of our knowledge, this is the sixth case reported in the literature and it is concordant with other published studies regarding clinical presentation and poor patient prognosis.

## Conclusions

Therefore, primary angiosarcomas of the male breast are extremely rare. This is the sixth case published in the literature and it is in agreement with other studies in the literature concerning clinical presentation and poor prognosis.
